# A Cross-Sectional Analysis of Ambulatory Oncology Experience by Treatment Intent

**DOI:** 10.3390/curroncol28010013

**Published:** 2020-12-18

**Authors:** Linda Watson, Siwei Qi, Eclair Photitai, Andrea DeIure

**Affiliations:** Applied Research and Patient Experience, Cancer Research and Analytics, Cancer Care Alberta—Alberta Health Services, Calgary, AB T2S 3C3, Canada; siwei.qi@ahs.ca (S.Q.); eclair.photitai@ahs.ca (E.P.); andrea.deiure@ahs.ca (A.D.)

**Keywords:** ambulatory oncology patient satisfaction survey, AOPSS, oncology, ambulatory, cancer, patient experience, patient satisfaction, palliative care, goal of treatment, treatment intent, quality improvement, person-centred care, patient-centred care, quality of life

## Abstract

The Ambulatory Oncology Patient Satisfaction Survey (AOPSS) is a standardized instrument to assess the overall cancer patient experience. This study retrospectively investigated differences in care experiences and satisfaction among ambulatory oncology patients who self-identified as receiving outpatient therapies for curative intent or for symptom or disease control. This cross-sectional study analyzed data from the AOPSS collected between February and April 2019 within the provincial cancer program in Alberta, Canada. There were 2104 participants who returned the survey, representing a 52.7% response rate. This nationally validated survey gathers patient care experiences and satisfaction across six domains of person-centred care. Treatment intent was characterized by adding a new “goal of treatment” question. Statistical analysis was performed using Mann–Whitney U tests and analysis of covariance (ANCOVAs). Cancer patients’ treatment goals were found to be significantly associated with key patient characteristics like age, sex, tumour group, and the locations where they received care. Patients whose self-identified goal of treatment was to cure their cancer reported significantly higher levels of satisfaction and a more positive experience in five out of the six person-centred care domains. Results identify marked differences in satisfaction and experience between these two patient groups even though they both received care in the same ambulatory environments. A better understanding of the experience and satisfaction of non-curative cancer patients could allow for a more holistic and supportive approach to patient care. In addition, an early palliative approach to care is recommended for improved patient outcomes.

## 1. Introduction

Patients with cancer are often confronted with difficult decisions regarding treatment options and their associated side effects [1]. For these patients, understanding the specific goal of their treatment plays a pivotal role in their cancer journey [1]. If realistic treatment goals are not discussed and established, undo suffering may occur [2]. On the other hand, when clinicians understand the patients’ goals of treatment, they are better able to tailor their care delivery to each patient’s unique needs [3] thus leading to a more person-centred approach to care and improved patient experience and satisfaction. Person-centred care is about treating people with cancer as individuals and establishing a partnership between health care providers, patients, and their loved ones [4]. This concept is also supported by Alberta Health Services’ Patient First Strategy, which recommends that patients (and family/primary support persons as the patient chooses) engage as full partners in their care, collaborating as essential members with their health care teams [5]. This approach to care promotes a shared holistic understanding of cancer patients’ needs and goals which is crucial to optimizing health processes and outcomes like improving the experience for patients in Alberta.

Although it makes intuitive sense that treatment goals may impact patient satisfaction and their experience of receiving care, previous studies have yet to report on the distinct treatment goals in large samples of cancer patient experience data. In response, we conducted a cross-sectional study of cancer patient experience with individuals who had received ambulatory cancer treatment in the past six months in Alberta, to explore the prevalence of their perceived goal of treatment. Moreover, we sought to investigate the relationship between their treatment goals, their experience, and their satisfaction. To our knowledge, no previous large-scale study has examined the impact of treatment goals on cancer patients’ experience and satisfaction. We hypothesized that cancer patients whose treatment goals were to cure their cancer would report a more positive experience and higher level of satisfaction than those whose treatment goals were to control their symptoms or cancer progression.

## 2. Methods

### 2.1. Design

This cross-sectional study used the survey data collected by the Ambulatory Oncology Patient Satisfaction Survey (AOPSS), distributed and collected between February 2019 and April 2019 across all 17 cancer centres in Alberta, Canada.

### 2.2. Participants

The random sample included a selection of Albertans who had a cancer diagnosis and had received at least one ambulatory cancer treatment in the last 6 months. Cancer treatment was classified as any systemic or oral therapy (e.g., chemotherapy or immunotherapy) and/or radiation therapy. The sample included patients from the two large tertiary, four regional and 11 community cancer centres in the province. Patients were linked to the sample based on what facility they received their cancer treatments at. For example, a patient living in Drumheller (a small town northeast of Calgary) may have seen an oncologist for their consultations in either Calgary (tertiary) or Red Deer (regional), but would have likely received their actual treatments in Drumheller, and therefore this patients satisfaction data would be linked to the Drumheller community cancer centre. For the purposes of this study, we classified tertiary satisfaction data as “urban” and regional and community satisfaction data as “rural and remote”.

A total of 4039 surveys were distributed, 45 (1.1%) of the surveys were non-deliverable due to address change or other reasons. The total number of participants who completed and returned the survey was 2104 resulting in a 52.7% response rate.

### 2.3. Procedure

In the AOPSS survey cycle, mail-outs were launched in February 2019 and closed 12 weeks later in April. Patients received a package containing an information sheet, a self-addressed and stamped return envelope, and a paper survey. Patients who had not yet responded to the survey then received a reminder package in March 2019 reminding them to complete the survey. This project was conducted in compliance with the Helsinki Declaration and the Alberta Research Ethics Community Consensus Initiative (ARECCI) ethical guidelines for quality improvement and evaluation. After screening with the ethical principles established by ARECCI, it was classified as quality improvement and the need for full REB review was waived. No harm was anticipated or actually reported in relation to this project.

### 2.4. Measures

The AOPSS experience measure was first introduced in 2002, was developed and validated nationally by the National Research Corporation (NRC) in 2003 and has been adopted by many of the cancer jurisdictions in Canada (NRC, 2003) [6]. The AOPSS addresses the patient experience across multiple types of cancer care experiences, including surgery, ambulatory chemotherapy, radiation therapy and supportive care. Forty-four core items are used to construct six domains of person-centred care to understand patients’ experience: Physical Comfort; Information, Communication & Education; Coordination & Integration of Care; Respect for Patient Preferences; Emotional Support; and Access to Care. Domain scores represent the average of responses to specific questions which fall under each domain and are transformed linearly to a 0%-to-100% scale following scoring guidance from NRC (NRC, 2003) [6]. Overall satisfaction with care is also assessed in the survey by the following three questions: (1) “Did you feel that your care providers did everything they could to treat your cancer?” (2) “Overall, how would you rate the quality of care in the past 6 months?” (3) “Would you recommend the health care providers to your family and friends?”

To answer our research questions, we added a question in the AOPSS which asked respondents about the goal of their treatment. This question was adopted from the Goal of Care (GOC) model proposed by Thomas et al. in 2014 [7]. In this model, GOC was classified into three categories: (1) curative or restorative, (2) palliative and (3) terminal [7]. We modified this model slightly and the additional question was worded as follows: “What is the goal of your treatment?” The respondents were provided with the following statements to choose from: (i) “To cure my cancer”, (ii) To control my symptoms or cancer progression”, and (iii) “I do not know”.

### 2.5. Statistical Analyses

Descriptive statistics were applied for sociodemographic and disease-specific data. Chi-squared tests (for categorical variables) were used to test for potential differences between cancer treatment goals and sociodemographic and disease-specific variables. Analysis of covariance (ANCOVA) was conducted to assess group differences on the scores of the six domains of person-centred care, which were construed as continuous variables, to eliminate the effects of the confounding factors. Mann–Whitney U tests with Bonferroni adjusted alpha were conducted to correct for multiple comparisons and to test for potential group differences on the overall satisfaction (ordinal variables). Data were exported into SPSS Version 25.0 (Chicago, IL, USA) for analysis and statistical significance was set a priori at *p* < 0.05.

## 3. Results

### 3.1. Sample

Of the 2104 participants who completed the survey, 1174 (55.8%) were women and 990 were men (44.2%). The mean age of this cohort was 65.6 years (SD = 12.0) and more than half (57.3%) were aged 65 or older. The most common tumor group was hematology (25.3%), followed by breast (23.1%), gastrointestinal (14.0%), genitourinary (13.4%) and intrathoracic (11.5%). More than half of the participants held a postsecondary degree (54.7%) and 57.3% of respondents had received cancer care primarily at a tertiary cancer centres.

### 3.2. Patient Treatment Goals and Patient Characteristics

The vast majority of respondents (*n* = 1996, 94.9%) answered the question regarding the goal of their treatment with only 108 (5.1%) skipping the question. Of the 1996 respondents who answered this question, a slightly higher percent of patients reported visiting cancer facilities for the purpose of curing their cancer (*n* = 1026, 51.4%) and 919 respondents reported their treatment goal was to control their symptoms or cancer progression (46.0%). Fifty-one patients (2.6%) chose “I do not know”, and this response was excluded from the data analysis due to the small sample size. Table 1 presents the distribution of sociodemographic and disease-specific variables by patient treatment goals for the two groups. Patients’ treatment goals were found to be significantly associated with age groups, χ^2^ (4, *n* = 1945) = 36.2, *p* < 0.01; with sex, χ^2^ (1, *n* = 1945) = 4.85, *p* < 0.05; with tumor groups, χ^2^ (12, *n* = 1945) = 207.1, *p* < 0.01 and with the cancer centres where they received treatments, χ^2^ (2, *n* = 1945) = 13.0, *p* < 0.01. No significant association was noted between the treatment goals and the education levels, *p* > 0.05.

### 3.3. Six Dimensions of Person-Centred Care and Goals of Treatment

The normality was tested by Shapiro–Wilk test and all the alphas were >0.05. A series of ANCOVAs were conducted to determine the differences in domain scores between respondents with respect to different treatment goals. The assumption of homogeneity of variances were not violated in that the alphas of Levene’s test of equality ranged from 0.031 to 0.146 [8]. After controlling for the significant covariates (age, sex, tumour group, and cancer centres), results demonstrated that treatment goals were significantly associated with five domains: Access to Care, F (1, 1162) = 4.99, *p* < 0.05; Emotional Support, F (1, 1671) = 8.48, *p* < 0.01; Respect for Patient Preferences, F (1, 1923) = 13.2, *p* < 0.01; Coordination & Integration of Care, F (1, 1922) = 10.7, *p* < 0.01 and Information, Communication & Education, F (1, 1900) = 16.7, *p* < 0.01 (Table 2). Cancer patients who indicated that their treatment goal was to cure their cancer scored significantly higher than those whose treatment goal was to control their symptoms or cancer progression in five domains (Table 2). No significant association was noted between respondents’ treatment goals and the domain of Physical Comfort, *p* > 0.05.

### 3.4. Overall Satisfaction towards Quality of Care and Goals of Treatment

Reponses to the three overall satisfaction questions were ordinal and not normally distributed. Consequently, Mann–Whitney U tests with a Bonferroni adjusted alpha level of 0.017 were applied to compare the three overall satisfaction ratings between the two groups. These data demonstrate that the group whose treatment goal was to cure their cancer rated significantly higher on their response to the statement “Did you feel that your care providers did everything they could to treat your cancer?” (U = 434,936, *p* < 0.01) when compared to the group whose treatment goal was to control their symptoms or cancer progression. The satisfaction level related to overall quality of care in the “to cure cancer” group was also significantly higher than the “to control symptoms or cancer progression” group (U = 418, 472, *p* < 0.01). Table 3 lists the statistics.

## 4. Discussion

When measuring patient experience and satisfaction, it is generally uncommon to ask cancer patients about the intent of their treatment and how it relates to the anticipated outcome of their disease. When examining Patient Reported Experience Measures (PREMs) like the AOPSS, if we do not seek to understand the intent of patients’ treatment, then it is impossible to understand the difference that treatment intent makes to the patient experience. However, identifying the distinct treatment goals of cancer patients is a logical step in quality improvement as it can help to effectively reframe treatments from the patient’s perspective [9] and allow for more targeted quality improvement efforts within the cancer care program. Furthermore, discussions with cancer patients regarding their treatment goals could prevent the intensive and even unnecessary health system utilization near their end of life [10] and allow for the integration of specialized supports such as, an early palliative approach to care that has been shown to optimize quality of life (QOL) and improve survival [11]. Herein, we explored and reported on the prevalence of treatment goals among cancer patients who had received ambulatory cancer treatment within a six-month period. To our knowledge, no previous studies had reported on this aspect in a large cohort of cancer patients.

These data reported above (Table 1) indicate that the majority of patients who responded to the AOPSS survey (94.4%), had an awareness about whether their cancer was curable vs. non-curable. There were 919 patients who reported their treatment goal was to control their symptoms or cancer progression (46.0%) and only fifty-one patients (2.6%) chose the response “I do not know”. Treatment goals were found to be significantly associated with certain patient characteristics such as age, sex, and tumor group. However, we also found that, when compared with cancer patients whose goal was to cure their cancer, those whose goal was to control their symptoms or cancer progression scored lower in all six dimensions of person-centred care and five were at statistically significant levels. In addition, this same group of patients rated lower in three overall satisfaction questions and two were at a statistically significant level. Altogether, it can be concluded that cancer patients who reported that their treatment goal was to control their symptoms or cancer progression were less satisfied with their care, and consequently, could benefit from targeted efforts to improve their care experiences.

In 2017, Arora [12] reported that throughout a patients’ cancer journey, there is a need for ongoing support to understand complex medical information and adjust to and cope with changes in their diagnosis and varying responses to treatments. Our findings further support this statement as we found that patients whose goal was to control their symptoms or cancer progression scored lower in informational and emotional aspects of their care and likely could have benefited from more supports in these areas. Our findings have important implications as they suggest that health care providers should evaluate their patients’ perceptions of their goals of treatment and then provide services accordingly. In addition, there are no question(s) in our current AOPSS that addresses patients’ access to or satisfaction with palliative care resources. Adding in a question of this nature could help providers and inform the larger health care system about how to ensure that patients who have received a non-curable cancer diagnosis could be supported faster and more appropriately. As summarized by Schottenfeld et al. [13], the key component of high-quality care is to actively seek and appropriately respond to patients’ preferences and goals. Kendall et al., [14] stressed that a detailed understanding of the varied experiences of people with different goals of care can help policymakers and clinicians design and deliver more appropriate and person-centered services.

Many advances have occurred in cancer therapeutics over the past two decades, allowing more patients with advanced cancer to live longer than ever before [15]. However, despite these advancements, patients with cancer continue to experience significant distress from symptoms of their disease and treatment side effects which often go underdiagnosed and undertreated [16], and this is more pronounced in patients with advanced cancer receiving treatments to control their disease progression [17]. While the management of symptoms and distress is viewed as an essential component of high-quality cancer care, with the increased volume of patients receiving treatments to control their advanced cancer, it is becoming more and more challenging for the oncology team to identify and address the needs of this patient population comprehensively [18]. As a result, early palliative care involvement alongside standard oncology care is now widely recommended [19].

In addition, there is strong evidence to suggest that the integration of a more holistic and supportive approach to care may be advantageous for advanced non-curative cancer patients and could even improve their QOL. The overall goal of supportive care is to prevent or treat the symptoms of a disease and the psychological, social, and spiritual problems related to that disease or its treatment, as early as possible [20]. In a study by Rummans et al. (2006) [21], advanced cancer patients were randomly assigned to either an eight-session structured multidisciplinary intervention arm which addressed five domains of QOL including cognitive, physical, emotional, spiritual, and social functioning or a standard care arm. They found that patients who were in the intervention sessions maintained and improved their QOL during radiation therapy, whereas patients who were in the standard care arm experienced a significant decrease in their QOL [21]. In addition to ensuring that patients with advanced disease are connected to palliative care resources, depending on the patients’ individual needs, it is also important to ensure they have access to interprofessional care providers like social workers, psychologists, physiotherapists, occupational therapists, dietitians, pharmacists, spiritual care workers and home health care aids as their needs evolve over time [22]. Interprofessional involvement is correlated with earlier conversations about preferences, values, and plans of care between patients’, their families, and the care team [23]. An interprofessional model of care has also been linked to improvements in the overall experiences of care for patients with advanced cancer [23]. Therefore, integrating a more interdisciplinary and holistic approach to care may be a relevant area for quality improvement to improve advance cancer patients’ levels of satisfaction and quality of life.

One other interesting finding from these data reported above is the differences in patient experience between urban and rural and remote areas. Previous studies have reported on this same comparison, for example, Katz et al. [24] and Mollica et al. [25] reported that cancer patients who resided in urban areas (vs rural and remote) rated their care significantly lower, but Charlton (2015) concluded patients tend to have more challenges and lower satisfaction levels in rural areas [26]. Our results shed light on this disparity, as previous AOPSS data revealed higher levels of satisfaction in rural and remote patients, but in this particular analysis, we also found that patients treated in rural and remote settings were more likely to report their treatment intent as curative. Therefore, treatment intent should be considered when analyzing overall satisfaction data in different geographical areas, as it may provide opportunities to improve the experience of patients who are receiving treatments to control the progression of their disease.

The major strength in this study is the large and randomly selected sample size, which ensures the generalizability to other cancer patient populations. A potential limitation is that we used a single question to assess the patients’ goals of treatment and a more in-depth qualitative component could provide additional insights. We also acknowledge that the study was limited by its retrospective nature, which restricted our selection of potential confounders when we conducted the between-subjects comparison. In addition, we could not authenticate if patients’ perceptions of their prognosis were accurate, as patients sometimes hold inaccurate perceptions of their prognosis and treatment intent [27]. Lastly, in the future, it may be important to explore satisfaction levels of participants who answered “I don’t know” to the question about the intent of their treatment as this could support the argument that patients feel more satisfied with their care when they clearly understand the intent of their cancer treatments.

## 5. Conclusions

Understanding a patient’s treatment goals in the context of their cancer allows the care team to align the care provided with what is most important to the patient [28]. Discussions about goals of treatment can help clinicians to better match recommended treatments to the patient’s unique situation and integrate additional symptom management and supportive care resources as needed, thereby improving their experience and satisfaction, leading to better QOL. Findings from this study can be utilized to identify gaps in patient experience for quality improvement and to serve as benchmarks to triage cancer patients for programmatic interventions such as a more holistic, supportive approach to patient care, involvement of the interprofessional team, and an early connection to the palliative care team. More cancer agencies should consider adding the question “what is the intent of your treatment” to standard patient experience measures as it allows insight into how many patients may require additional targeted support based on their treatment intent, leading to programmatic capacity in symptom management, supportive care resources, and palliative care.

## Figures and Tables

**Table 1:** Distribution of sociodemographic and disease-specific variables by patient treatment goals.

	Treatment Goals		
	To Cure Cancer (*n* = 1026)	To Control Symptoms (*n* = 919)	*p*
**Age Group**			0.000
18–34	22 (2.1%)	9 (1.0%)	
35–44	56 (5.5%)	21 (2.3%)	
45–54	123 (12.0%)	78 (8.5%)	
55–64	301 (29.3%)	235 (25.6%)	
65 and over	524 (51.1%)	576 (62.7%)	
**Sex**			0.028
Male	598 (58.3%)	490 (53.3%)	
Female	428 (41.7%)	429 (46.7%)	
**Tumour Groups**			0.000
Breast	332 (32.4%)	134 (14.6%)	
Gastrointestinal	153 (14.9%)	115 (12.5%)	
Genitourinary	162 (15.8%)	103 (11.2%)	
Gynecology	70 (6.8%)	34 (3.7%)	
Hematology	167 (16.3%)	317 (34.5%)	
Intrathoracic	63 (6.1%)	155 (16.9%)	
Other *	79 (7.7%)	61 (6.6%)	
**Educational Level**			0.493
Grade 9 or less	69 (6.7%)	60 (6.5%)	
Some high school but did not graduate	120 (11.7%)	116 (12.6%)	
High school graduate	269 (26.2%)	215 (23.4%)	
College, trade, or technical school	307 (29.9%)	301 (32.8%)	
University undergraduate degree	136 (13.3%)	104 (11.3%)	
Post university/graduate education	84 (8.2%)	86 (9.4%)	
Not answered	41 (4.0%)	37 (4.0%)	
**Cancer Centres**			0.001
Tertiary	589 (57.4%)	523 (56.9%)	
Regional	315 (30.7%)	327 (35.6%)	
Community	122 (11.9%)	69 (7.5%)	

* Other included: CNS, Endocrine, Head and Neck, Melanoma, Non melanoma skin, Other Malignant and Sarcoma.

**Table 2:** Six dimensions of person-centred care and goals of treatment.

	To Cure Cancer		To Control Symptoms					
	EMM *	SE **	EMM	SE	*df*	F	*p*	*η_p_*^2^ ***
Access to Care	75.2%	1.1%	71.8%	1.3%	1, 1162	4.99	0.026	0.004
Coordination & Integration of Care	70.4%	1.1%	66.0%	1.2%	1, 1922	10.7	0.001	0.006
Emotional Support	57.4%	1.3%	52.7%	1.4%	1, 1671	8.48	0.004	0.005
Information, Communication & Education	68.9%	1.2%	62.9%	1.3%	1, 1900	16.7	0.000	0.009
Physical Comfort	75.5%	1.9%	73.3%	2.2%	1, 899	2.93	0.087	0.003
Respect for Patient Preferences	83.6%	0.9%	79.6%	1.0%	1, 1923	13.2	0.000	0.007

* EMM: Estimated Marginal Mean. ** SE: Standard Error. *** *η_p_*^2^: Partial Eta Squared.

**Table 3:** Overall Satisfaction.

	To Cure Cancer		To Control Symptoms			
	*n*	Mean Rank	*n*	Mean Rank	*U*	*p*
Providers Did Everything to Treat Cancer	1020	995.1	911	933.4	434,936	0.000
Rate ambulatory care past 6 months	1019	1000.3	904	915.4	418,472	0.000
Would recommend providers	1019	976.1	909	951.5	451,287	0.048
